# Anthropometric data quality assessment in multisurvey studies of child growth

**DOI:** 10.1093/ajcn/nqaa162

**Published:** 2020-07-16

**Authors:** Nandita Perumal, Sorrel Namaste, Huma Qamar, Ashley Aimone, Diego G Bassani, Daniel E Roth

**Affiliations:** Department of Global Health and Population, Harvard TH Chan School of Public Health, Boston, MA, USA; Centre for Global Child Health, Hospital for Sick Children, Toronto, Ontario, Canada; The DHS Program, ICF, Rockville, MD, USA; Centre for Global Child Health, Hospital for Sick Children, Toronto, Ontario, Canada; Dalla Lana School of Public Health, University of Toronto, Toronto, Ontario, Canada; Centre for Global Child Health, Hospital for Sick Children, Toronto, Ontario, Canada; Dalla Lana School of Public Health, University of Toronto, Toronto, Ontario, Canada; Centre for Global Child Health, Hospital for Sick Children, Toronto, Ontario, Canada; Department of Pediatrics, Hospital for Sick Children, Toronto, Ontario, Canada; Department of Pediatric Medicine, Department of Nutritional Sciences, and Institute of Health Policy, Management and Evaluation, University of Toronto, Toronto, Ontario, Canada

**Keywords:** anthropometry, Demographic and Health Surveys, data quality, height-for-age *z* score, weight-for-height *z* score, stunting, wasting

## Abstract

**Background:**

Population-based surveys collect crucial data on anthropometric measures to track trends in stunting [height-for-age *z* score (HAZ) < −2SD] and wasting [weight-for-height *z* score (WHZ) < −2SD] prevalence among young children globally. However, the quality of the anthropometric data varies between surveys, which may affect population-based estimates of malnutrition.

**Objectives:**

We aimed to develop composite indices of anthropometric data quality for use in multisurvey analysis of child health and nutritional status.

**Methods:**

We used anthropometric data for children 0–59 mo of age from all publicly available Demographic and Health Surveys (DHS) from 2000 onwards. We derived 6 indicators of anthropometric data quality at the survey level, including *1*) date of birth completeness, *2*) anthropometric measure completeness, *3*) digit preference for height and age, *4*) difference in mean HAZ by month of birth, *5*) proportion of biologically implausible values, and *6*) dispersion of HAZ and WHZ distribution. Principal component factor analysis was used to generate a composite index of anthropometric data quality for HAZ and WHZ separately. Surveys were ranked from the highest (best) to the lowest (worst) index values in anthropometric quality across countries and over time.

**Results:**

Of the 145 DHS included, the majority (83 of 145; 57%) were conducted in Sub-Saharan Africa. Surveys were ranked from highest to lowest anthropometric data quality relative to other surveys using the composite index for HAZ. Although slightly higher values in recent DHS suggest potential improvements in anthropometric data quality over time, there continues to be substantial heterogeneity in the quality of anthropometric data across surveys. Results were similar for the WHZ data quality index.

**Conclusions:**

A composite index of anthropometric data quality using a parsimonious set of individual indicators can effectively discriminate among surveys with excellent and poor data quality. Such indices can be used to account for variations in anthropometric data quality in multisurvey epidemiologic analyses of child health.

## Introduction

Reductions in the global burden of malnutrition are central to the Sustainable Development Goals, which include specific targets related to stunting [height-for-age *z* scores (HAZ) < −2SD of the population median], wasting, and overweight [weight-for-height *z* scores (WHZ) < −2SD or >2SD of the population median, respectively] in children <5 y of age (1). Tracking progress toward global goals and identifying high-priority areas for investments are based on country-level prevalence estimates and trends in child malnutrition in low- and middle-income countries (LMICs) (2).

The Demographic and Health Surveys (DHS) Program conducts population-representative surveys in LMICs, including anthropometric data for children 0–59 mo of age in addition to other measures of health and development (3). These surveys are used to estimate and compare the nutritional status of young children within and between countries, to monitor secular trends, and to measure responses to public health interventions (4, 5). However, the validity and reliability of survey-based metrics of child nutritional status depend on the quality of the anthropometric data (6–8). In the context of multisurvey analyses, accounting for variability in anthropometric data quality is particularly important because the quality of anthropometric data is unlikely to be uniform across surveys (6).

Anthropometric data quality may be affected by survey design (e.g., sampling strategy, questionnaire design, and measurement tools), implementation (e.g., nonresponse rate, management of field operations, staff training in data collection and anthropometry measurement, and method of data entry), and data processing procedures (6, 8, 9). Several indicators have been used to assess anthropometric data quality including the pattern of age heaping (10), missingness of data on child height (11), proportion of biologically implausible values (6), misreporting of month of birth (MOB) for age estimation (12), and effect of random error (7). Whereas examining several individual indicators is informative for assessing various dimensions of quality within a single survey, for multisurvey analyses, a single aggregate measure of relative anthropometric data quality, which combines several data quality indicators, would better enable researchers to account for heterogeneity in the quality of anthropometric data collected across countries and over time.

In this study, we describe the development of composite indices of anthropometric data quality for use in multisurvey analysis of child health and nutritional status. The indices enable a relative assessment of the robustness of data underlying the estimation of metrics of HAZ (e.g., mean HAZ or stunting prevalence) or WHZ (e.g., mean WHZ or wasting or overweight prevalence) across multiple surveys.

## Methods

### Data source

We used individual-level data from all available Phase IV–VII DHS conducted since January 2000 and the public release of data as of January 2019. The rationale for the inclusion criteria was to ensure consistency in the sampling frame, because anthropometric measures in previous DHS phases (I–III) were collected only from the children of eligible women who were interviewed in each household. In subsequent DHS phases, all children in the surveyed households were eligible for anthropometric assessment. Surveys were retained in the present analysis even if anthropometry data were excluded from DHS final reports owing to data quality issues (i.e., surveys conducted in Benin in 2012 and in Jordan in 2007).

### Data quality indicators

We identified 7 indicators for HAZ and 4 indicators for WHZ data quality derived from anthropometric measures collected in population-based surveys per recommendations by the WHO/UNICEF Anthropometry Data Quality Working Group and research studies (12, 13). There are fewer indicators related to WHZ because information related to child age is not used to derive WHZ (only weight and height measures are needed). Four domains of anthropometric data quality are reflected by these indicators: *1*) partial or incomplete information for date of birth and anthropometry measurement, *2*) digit preference for height and age, *3*) bias in age-reporting, and *4*) dispersion and extreme values in the HAZ and WHZ distributions. Each data quality indicator was estimated at the survey level and is described in detail below.

#### Date of birth completeness

Date of birth completeness was defined as the percentage of children 0–59 mo with at least complete month and year of birth. Birth dates with missing day of birth were not considered to be incomplete because the 2006 WHO growth standards allow missing day of birth to be imputed (14).

#### Completeness of anthropometry measurement

Anthropometric measurement completeness was defined as the percentage of children 0–59 mo with height and weight data recorded. Participants that refused, were not present, or were not measured for another reason were considered incomplete.

#### Digit preference for height and age

Digit preference is defined as a tendency for certain digits to appear more often than expected by chance. Rounding or other forms of unevenness in the distributions of the last digit for height would suggest lack of care by the data collector or fabrication of data; for age, it is usually the result of respondents not knowing their child's date of birth and/or insufficient probing by the data collector. Digit preference for weight was not calculated because in many DHS weight is recorded to the hundredth decimal place but rounded to the tenth place in the publicly available data sets. The index of dissimilarity was used to numerically characterize digit preference for height and age separately. The index of dissimilarity is calculated as the sum of the actual distribution percentages of height (or age) minus the expected distribution percentages divided by 2 (Equation 1) (13):

(1) \begin{eqnarray*} &&{\text{Index of dissimilarity }}\\ && = {\rm{\Sigma abs\ }}\left( {{\rm{actual\ percentage}} - {\rm{expected\ percentage\ }}} \right) /2 \end{eqnarray*}

The index of dissimilarity is interpreted as the proportion of data that would need to be shifted to align with the expected percentage distribution of digit preference for height (or age). It is the measure of digit preference recommended by the WHO/UNICEF Anthropometry Data Quality Working Group and is similar to measures that have been used previously, such as Myers’ blended index (15). The digit to the right of the decimal place, on a centimeter scale, is used to examine digit preference for height. For age, there are several potential age patterns that are indicative of poor quality (e.g., different intervals for age in completed years, different intervals for age in completed months, calendar MOB). We used a consistent indicator of age preference across surveys—heaping at 6 mo of age intervals—to assess digit preference because this was expected to be a common pattern across surveys.

#### Absolute difference in HAZ by MOB

Although there may be potential seasonal patterns in the relationship between mean HAZ and MOB, there should be no sharp differences in mean HAZ by the MOB within a given birth year. In situations with poor date of birth information, however, discontinuities in estimates of mean HAZ by MOB are more likely to occur near the end/beginning of a calendar year. This is because children who are born early in the year are likely to be randomly assigned later birth months, and vice versa for children born later in the year. For example, a child who is erroneously recorded as being born earlier in the year than the true birth month is actually younger than reported and therefore likely to be assigned an inappropriately low HAZ for age. Therefore, surveys with larger differences in HAZ by MOB may indicate errors in age reporting, and thus biased HAZ estimates. The degree of bias in MOB reporting for each survey was assessed by the absolute difference between the mean HAZ values for January and December (12). The absolute difference in HAZ by MOB of reporting should be close to 0 if there are no systematic errors in age reporting.

#### Biologically implausible (“flagged” values)

We defined biologically implausible values for HAZ as those >6 SDs above or below and for WHZ as >5 SDs above or below the median *z* score of the reference population according to the WHO flagging convention (14). The WHO macro excludes children if their length is outside of the ranges of 45–110 cm or if their height is outside of the ranges of 65–120 cm when calculating weight-for-height *z* scores before flagging values outside of the plausible WHZ *z* score ranges (13). Thus, when calculating the percentage of implausible values for WHZ, out-of-range values were added to the numerator and denominator after applying the WHO macro software.

#### HAZ and WHZ dispersion

The width of the distributions of HAZ and WHZ may be described using the SD calculated after removal of flagged values based on the WHO flagging convention. The higher the SD above 1.0, the expected value for a Gaussian distribution, the more likely there is a data quality problem (14). However, because SD is a function of the HAZ/WHZ distributions itself, SD may be naturally increased as a result of heterogeneity of the survey population (15). HAZ SD also varies with age within surveys and there is an empirical association of mean HAZ with HAZ SD across surveys (16). As such, SD has limitations as a stand-alone indicator of survey quality.

### Statistical analysis

We applied the 2006 WHO standard (14) macro to the raw DHS data files to derive estimates for HAZ, WHZ, prevalence of stunting, and prevalence of wasting among children 0–59 mo of age. All measures and estimates were calculated for *de facto* children, defined as the members of the household 0–59 mo of age who slept in the household the previous night (17). Estimates for mean HAZ and WHZ and prevalence of stunting and wasting were derived using survey weights; however, data quality indicators did not account for the complex survey design given that the observed survey samples were of primary interest in assessing quality.

Each individual indicator of data quality was derived at the survey level and summarized across surveys. To examine collinearity between metrics, we estimated a matrix of Pearson correlations of mean HAZ (or mean WHZ), prevalence of stunting (or wasting), and each of the individual indicators of data quality for HAZ or WHZ, respectively. Principal component factor analysis (PCA) was used to generate composite data quality indices for HAZ and WHZ separately. PCA is a data reduction strategy used to generate an index which summarizes the largest variability in the data using the correlation matrix of a linear combination of variables in the first component (18). This strategy is well-suited for generating a composite index that explains the greatest total variance in the data based on the correlation matrix, producing standardized estimates of the amount of variance in the data explained by each indicator.

To generate the quality index for HAZ, we initially considered 7 data quality indicators estimated at the survey level in the PCA: *1*) proportion of observations with complete date of birth, *2*) proportion of observations with anthropometry measured, *3*) digit preference for height, *4*) digit preference for age, *5*) absolute difference in the mean HAZ by MOB, *6*) proportion of biologically implausible (flagged) values for HAZ based on the WHO criteria, and *7*) HAZ SD. However, digit preference for age demonstrated low variability across surveys (**Table 1**) and explained <5% of the total variance in the principal component (data not shown). In addition, age preference patterns likely differ between surveys and cannot be standardized across surveys without graphically examining the pattern within each survey. As such, we did not include this variable in the final index which included the remaining 6 data quality indicators for HAZ (6Q). After PCA, we generated a predicted factor index for the first component, which explained the largest variance based on the correlation matrix of individual variables (44% for HAZ and 60% of WHZ), and used the negative values of the predicted index (i.e., reversing the sign to improve interpretability) to compare relative anthropometric data quality across surveys. Lower values on the resulting 6Q HAZ data quality index reflect lower data quality and higher values reflect higher data quality. It is important to note that the index values cannot be interpreted in absolute terms in a manner that would enable comparisons with surveys not included in the present analyses; rather, the indices enable ranking or between-survey comparisons in relative terms among the included surveys.

**TABLE 1:** Summary of individual quality indicators considered for use in the development of anthropometric data quality indices for HAZ and WHZ^1^

	Summary statistics		
Data quality indicator	Median	IQR	Range (min, max)
Completeness of date of birth, %	99	98–100	80–100
Completeness of anthropometry measurement, %	96	93–98	70–100
Digit preference for height, index of dissimilarity, %	15	10–24	3.1–83
Digit preference for age at 6-mo intervals, index of dissimilarity, %	0.70	0.50–0.90	0–2.0
Biologically implausible (“flagged”) values for HAZ, %	1.6	0.72–2.9	0.03–12
Biologically implausible (“flagged”) values for WHZ, %	1.9	1.0–3.4	0.13–15
Absolute difference in mean HAZ by month of birth (December vs. January), *z* score	0.25	0.13–0.38	0.001–0.90
SD of HAZ, *z* score	1.59	1.41–1.78	1.07–2.47
SD of WHZ,^2^*z* score	1.30	1.18–1.43	0.99–2.17

1 *n* = 145 Demographic and Health Surveys. HAZ, height-for-age *z* score; WHZ, weight-for-height *z* score.

2 WHZ missing for Madagascar.

The data quality index for WHZ was generated based on 4 data quality indicators (4Q): *1*) proportion of observations with complete anthropometry measures, *2*) digit preference for height, *3*) proportion of biologically implausible (flagged) values based on the WHO criteria, and *4*) WHZ SD. We excluded individual data quality indicators that were related to age because age is not used in the derivation of WHZ. We further excluded indicators of dispersion for HAZ; however, digit preference for height was included because height measures are used when deriving WHZ. The WHZ 4Q quality index was otherwise developed using the same approach as for the 6Q HAZ quality index.

A mixed-effects model with a random intercept for country was used to estimate the proportion of variance in data quality indices attributable to differences among countries as opposed to within-country heterogeneity (i.e., across multiple surveys in the same country). All analyses were conducted using STATA 15.0 software (StataCorp) and the code was cross-checked by a second individual independently.

### Ethics statement

This was a secondary analysis of publicly available deidentified data which is exempt from ethical review at the Hospital for Sick Children Ethics Review Board and the ICF Review Board.

## Results

Characteristics of the individual indicators of anthropometric data quality across the 145 DHS overall and by world region are summarized in Tables 1 and **2**, respectively. Overall, completeness of date of birth (i.e., at least a month and year measured) and of anthropometry measurement were high, ranging from ∼70% to 100% across surveys for both indicators (Table 1). The index of dissimilarity is expected to be close to 0 when there is little to no evidence of digit preference. However, the index of dissimilarity for height was 15% overall, ranging widely from 3.1% to 83% (Table 1). The proportions of biologically implausible values for HAZ and WHZ were similar overall, although the median and the range of flagged values were higher for WHZ. Conversely, the median SD and range for HAZ were greater overall and across all surveys than for WHZ (Tables 1, 2). The absolute difference in HAZ by MOB of age reporting should be close to 0 if there is no systematic error in age reporting, but was 0.25 (in *z* score units) overall and up to 0.90 in Timor-Leste in 2009 (Table 1). There was substantial variability in all indicators of data quality based on the world region, with the greatest degree of heterogeneity observed among surveys in Sub-Saharan Africa where the majority of surveys were conducted (Table 2).

**TABLE 2:** Summary of individual indicators of data quality disaggregated by world region of Demographic and Health Surveys^1^

	East Asia and Pacific (*n* = 7)			Europe and Central Asia (*n* = 10)			Latin America and the Caribbean (*n* = 21)			Middle East and North Africa (*n* = 11)			South Asia (*n* = 13)			Sub-Saharan Africa (*n* = 83)		
Data quality indicator	Median	IQR	Min, max	Median	IQR	Min, max	Median	IQR	Min, max	Median	IQR	Min, max	Median	IQR	Min, max	Median	IQR	Min, max
Date of birth complete, %	100	99–100	99, 100	100	99–100	98, 100	99	98–99	94, 100	100	100–100	99, 100	99	97–100	94, 100	99	97–99	80, 100
Anthropometry measurement complete, %	97	92–98	89, 98	95	93–97	99, 100	95	93–98	82, 99	96	92–99	83, 99	95	90, 98	70, 99	96	94–98	74, 100
Digit preference height, index of dissimilarity, %	22	16–23	11, 38	23	13–38	8.5, 56	9.9	5.9–13	3.1, 24	18	14–41	11, 59	9.3	7.5–12	5.7, 34	15	12–25	4.5, 83
“Flagged” values for HAZ, %	1.8	1.2–4.4	0.41, 4.6	2.0	0.84–2.7	0.39, 4.8	0.48	0.14–0.69	0.03, 5.4	1.9	0.82–4.1	0.40, 4.4	1.4	0.70–2.0	0.29, 11.6	1.8	1.2–3.3	0.13, 11.2
“Flagged” values for WHZ,^2^ %	1.8	1.4–4.7	0.78, 7.7	3.0	2.0–4.0	0.81, 12	0.82	0.30–1.3	0.13, 7.9	3.0	1.0–5.0	0.61, 6.9	1.8	0.64–2.8	0.39, 11	2.1	1.3–3.5	0.17, 15
Absolute difference in HAZ by month of birth, *z* score	0.23	0.14–0.41	0.13, 0.90	0.19	0.06–0.38	0.03, 0.57	0.15	0.07–0.26	0.01, 0.50	0.16	0.12–0.37	0.06, 0.49	0.15	0.06–0.24	0.002, 0.32	0.30	0.18–0.43	0.001, 0.83
SD of HAZ, *z* score	1.47	1.40–1.98	1.33, 2.17	1.68	1.54–1.71	1.46, 2.31	1.34	1.20–1.46	1.07, 1.58	1.69	1.28–1.91	1.22, 2.16	1.41	1.38–1.68	1.34, 1.98	1.66	1.50–1.85	1.27, 2.47
SD of WHZ,^2^*z* score	1.22	1.09–1.64	1.08, 1.70	1.44	1.32–1.54	1.24, 1.93	1.11	1.02–1.19	0.99, 1.39	1.40	1.11–1.75	1.09, 1.80	1.16	1.12–1.38	1.07, 1.64	1.32	1.22–1.43	1.05, 2.17

1 *n* = 145 overall. HAZ, height-for-age *z* score; WHZ, weight-for-height *z* score.

2 WHZ missing for Madagascar.

Pairwise correlations between the 6 individual data quality indicators of HAZ or WHZ were relatively weak (most <0.30), with the exception of the correlations between the flagged values and the SDs of HAZ and WHZ which were 0.79 and 0.86, respectively (**Table 3**, **Supplemental Figures 1** and **2**). The lack of strong correlations between individual data quality indicators suggests that each indicator used in the PCA reflects a different aspect of anthropometric data quality. Indicators of dispersion (i.e., SD of HAZ/WHZ and percentage of flagged values) had the highest factor loadings followed by digit preference for height in the data quality indices for HAZ and WHZ (**Table 4**).

**TABLE 3:** Correlation coefficients for mean HAZ and WHZ as well as the prevalence of stunting and wasting among children <5 y of age, data quality indices, and 6 individual metrics of anthropometric data quality across 145 Demographic and Health Surveys^1^

Data quality indicator	Mean HAZ	Stunting %	Data quality index for HAZ (6Q)	Mean WHZ	Wasting	Data quality index for WHZ (4Q)	Date of birth complete	% Measured	Digit preference for height	Flagged values for HAZ	Flagged values for WHZ	Difference in HAZ by MOB	HAZ SD	WHZ SD
Mean HAZ among children <5 y	1.00													
Stunting prevalence children <5 y	−0.96	1.00												
Data Quality Index HAZ (6Q)	0.12	−0.33	1.00											
Mean WHZ among children <5 y	0.42	−0.42	0.13	1.00										
Wasting prevalence children <5 y	−0.38	0.50	−0.51	−0.81	1.00									
Data Quality Index WHZ (4Q)	−0.02	−0.20	0.93	0.01	−0.46	1.00								
Completeness of date of birth	0.09	−0.12	0.51	0.10	−0.19	0.35	1.00							
Completeness of anthropometry	−0.13	0.10	0.33	0.01	−0.07	0.37	0.27	1.00						
Digit preference height	0.05	0.09	−0.67	0.04	0.24	−0.77	−0.24	−0.26	1.00					
Flagged values for HAZ	−0.17	0.36	−0.89	−0.11	0.46	−0.85	−0.35	−0.24	0.47	1.00				
Flagged values for WHZ	−0.02	0.23	−0.86	−0.03	0.45	−0.90	−0.32	−0.22	0.47	0.93	1.00			
Difference in HAZ by MOB	−0.11	0.22	−0.54	−0.14	0.25	−0.33	−0.09	0.03	0.13	0.37	0.35	1.00		
SD of HAZ	−0.16	0.40	−0.87	−0.17	0.59	−0.82	−0.28	−0.06	0.46	0.79	0.81	0.50	1.00	
SD of WHZ	−0.03	0.26	−0.88	−0.04	0.53	−0.92	−0.28	−0.13	0.60	0.81	0.86	0.40	0.92	1.00

1 HAZ, height-for-age *z* score; MOB, month of birth; WHZ, weight-for-height *z* score; 4Q, 4 data quality indicators; 6Q, 6 data quality indicators.

**TABLE 4:** Factor loadings of individual anthropometric quality indicators in principal component factor analyses using 145 Demographic and Health Surveys, conducted separately for HAZ and WHZ quality indices^1^

Data quality indicator	Factor loadings for 6Q HAZ index	Factor loadings for 4Q WHZ index
Completeness of date of birth, %	0.504	—
Completeness of anthropometry measurement, %	0.334	0.373
Digit preference for height, index of dissimilarity, %	0.665	0.768
Flagged values for HAZ, %	0.886	—
Flagged values for WHZ, %	—	0.896
Absolute difference in HAZ by month of birth, *z* score	0.534	—
SD of HAZ, *z* score	0.867	—
SD of WHZ, *z* score	—	0.924

1 HAZ, height-for-age *z* score; WHZ, weight-for-height *z* score; 4Q, 4 data quality indicators; 6Q, 6 data quality indicators.

**FIGURE 1:**
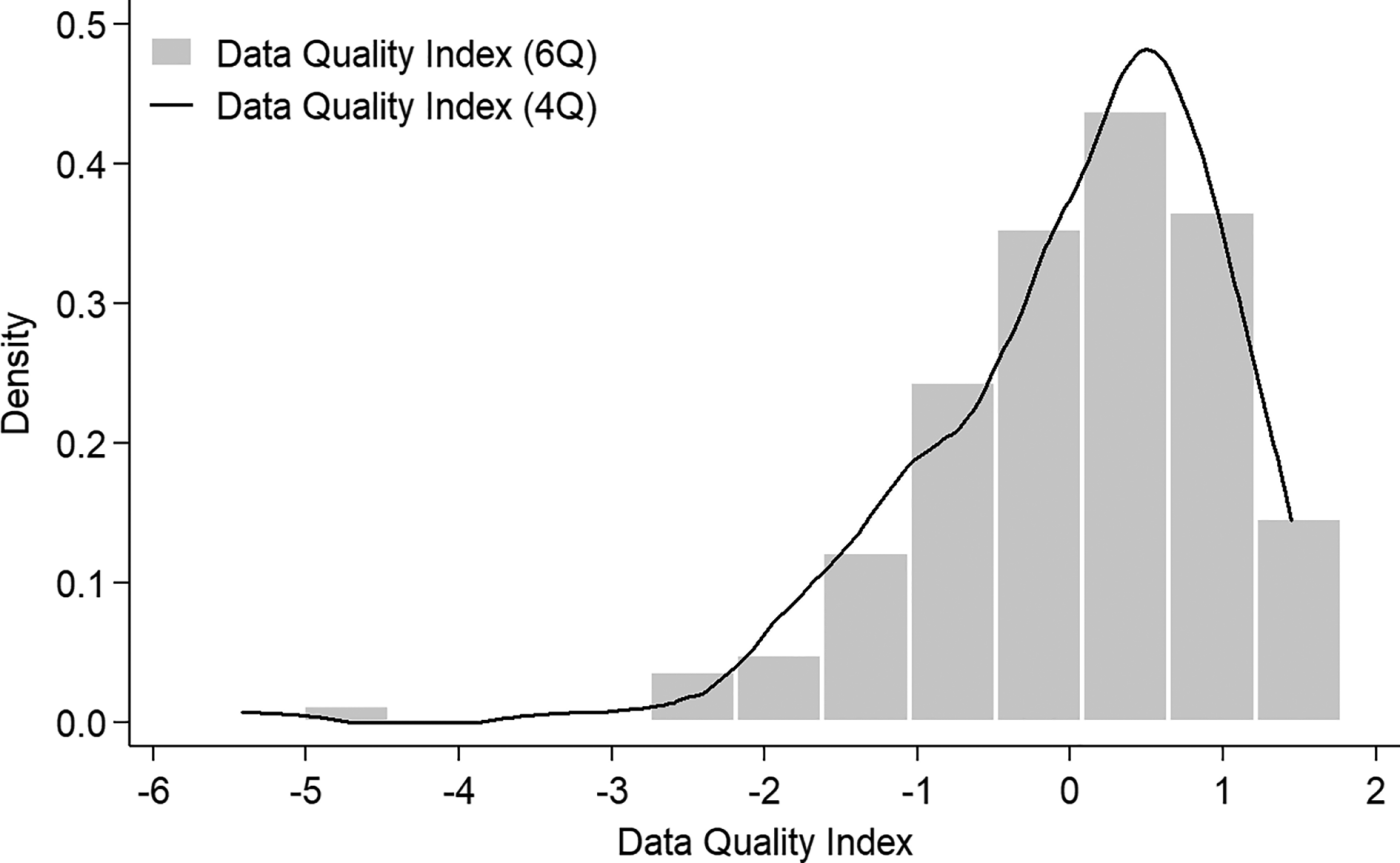
Distribution of anthropometric data quality index values in 145 Demographic and Health Surveys derived from principal component factor analysis based on 6 individual anthropometric quality indicators for height-for-age *z* score and 4 individual anthropometric quality indicators for weight-for-height *z* score. Lower index values reflect worse data quality. 4Q, 4 data quality indicators; 6Q, 6 data quality indicators.

**FIGURE 2:**
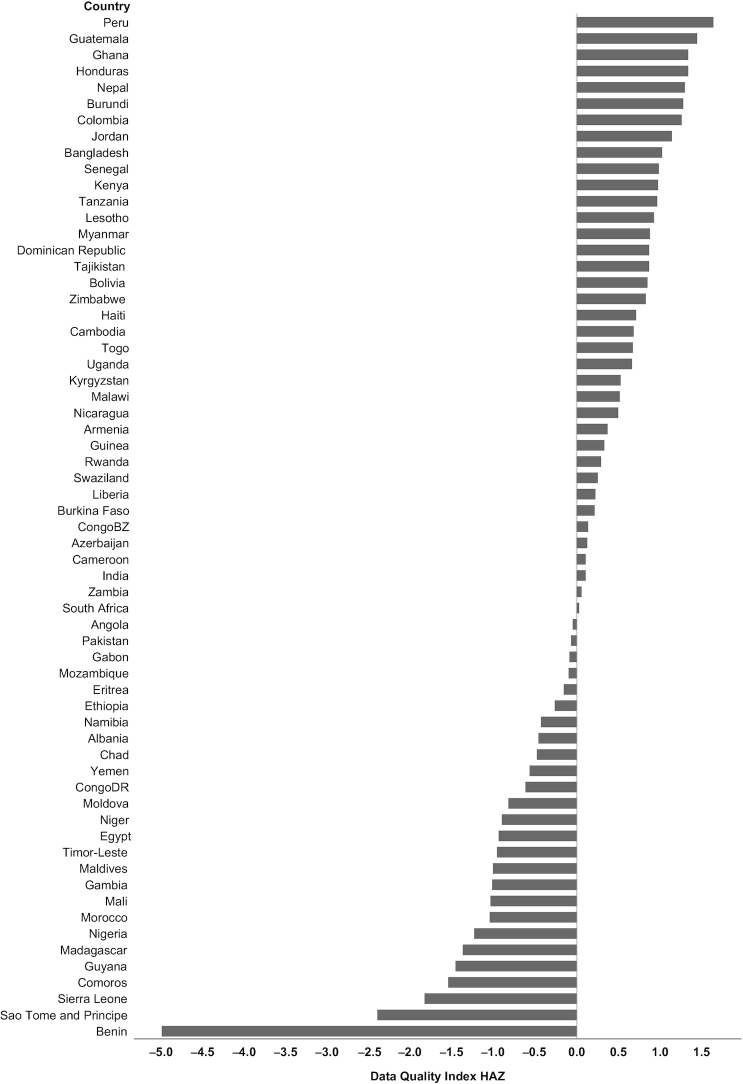
Country ranking of anthropometric data quality index values for HAZ (based on 6 individual anthropometric quality indicators) for the most recent Demographic and Health Survey for each country (*n* = 64). Greater index values indicate higher anthropometric data quality. HAZ, height-for-age *z* score.

The data quality index for HAZ ranged from −5.01 to 1.78 (**Figure 1**), and for WHZ from −4.34 to 1.47. To assess the validity of the data quality index, countries were ranked in descending order (from high to low quality) based on the data quality index for HAZ using the most recent survey (**Figure 2**). Peru had the highest data quality index in the most recent 2012 survey, with notable improvements in data quality in Peru occurring with each survey round since 2001 (**Supplemental Figure 3**). In contrast, the most recent survey for Benin in 2012 had the lowest index value across the 145 DHS. A decline in the quality of the anthropometry data was observed among Benin DHS from 2001 to 2012 (Supplemental Figure 3).

Similar rankings and changes in quality index values over time were observed when assessing data quality based on WHZ; however, there were notable differences in country rankings in the middle and lowest range of the index when compared with country rankings by HAZ data quality index (**Supplemental Figures 4** and **5**). Nonetheless, the correlation between the data quality indices for HAZ and WHZ was 0.93, indicating that surveys with higher data quality for HAZ also have a high data quality for WHZ (Table 3). This is not surprising given that 2 of the 6 metrics in the HAZ data quality index were also used for the data quality index for WHZ, and the 2 metrics unique to the data quality index for WHZ (proportion of flagged values and SD of WHZ) were each strongly correlated with flagged values and SD of HAZ (correlation coefficients of 0.93 and 0.92, respectively) (Table 3).

**FIGURE 3:**
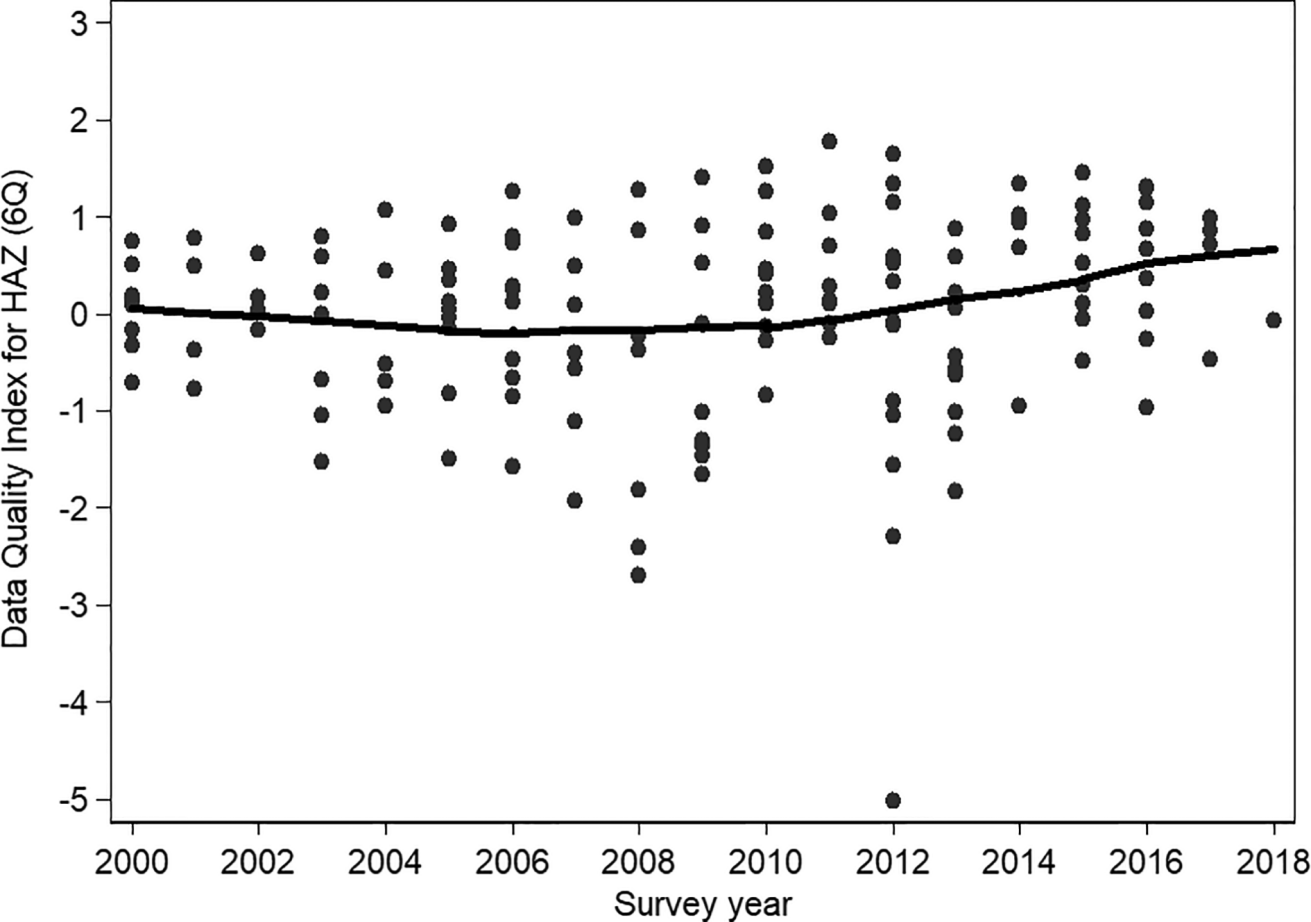
Scatter plot of anthropometric data quality index for HAZ over time in 145 Demographic and Health Surveys. Change in the data quality index over time is shown by a locally weighted smoothing spline. HAZ, height-for-age *z* score; 6Q, 6 data quality indicators.

The slightly upward-trending loess curve for 6Q data quality index values suggests an improvement in the quality of anthropometry data in DHS over time, particularly from ∼2008 to 2010 onwards (see **Figure 3** for HAZ and **Supplemental Figure 6** for WHZ); however, substantial heterogeneity in the anthropometric data quality still exists even in recent surveys. The intraclass correlation coefficients for the 6Q HAZ and 4Q WHZ data quality indices suggest that 56% and 48%, respectively, of the variance in the anthropometric data quality over time was attributable to variation between countries as opposed to within country.

## Discussion

We used a parsimonious set of individual indicators to derive composite indices to evaluate the relative anthropometric data quality of 145 DHS. The findings from this study demonstrate that a composite data quality index can effectively discriminate surveys with excellent anthropometric data quality from those with poor anthropometric data quality in the context of multisurvey analyses. Although the data quality index suggests that on average there may be some improvements in the overall quality of anthropometric data collection in DHS over time, there continues to be substantial heterogeneity in anthropometric data quality in surveys across countries. These indices can therefore be used to account for variability in anthropometric data quality across surveys or check the robustness of inferences related to metrics of child nutritional status and estimates of malnutrition across countries or over time. For example, the index can be used to assess robustness of inferences in multicountry and time-trend analyses by excluding surveys with the poorest relative anthropometric quality or by including the data quality index as a regression model covariate in sensitivity analyses. The high correlation between the HAZ and WHZ quality indices also suggests that a composite index, which includes individual indicators for quality of HAZ alone, captures issues in quality for other metrics of nutritional status as well.

The composite anthropometric data quality index may also be an effective tool to advocate for strengthening of rigorous collection of anthropometric data. For example, this anthropometric data quality index has already been used as part of The DHS Program's internal Survey Inception Tool, which is used to conduct an in-depth review of previous surveys in a country. It provides a snapshot of anthropometry quality over time within the country and the country ranking which can be used to inform recommendations on what can be done to improve data quality in the next survey round. The anthropometry data quality index has also been assessed for potential use during survey implementation for field monitoring purposes and to compare data quality between data collection teams (19). In large-scale surveys, where data are typically collected by several teams over a long duration, anthropometric data quality can be assessed in real time by examining data as they are being collected and identifying patterns that are indicative of poor quality (20). This information could be used to prioritize the retraining of the poorest-performing teams. Targeted investments to improve quality of survey data collection have been shown to improve anthropometric data quality (21). However, variations in quality across surveys are also likely to be related to contextual factors that hindered the successful implementation of a particular survey (e.g., conflict or political instability, geographically hard-to-reach populations).

Several previous studies have examined the effect of individual indicators of anthropometric quality on population-averaged metrics of child nutritional status (10–12). Methodological development in the systematic assessment of anthropometric data quality across multiple surveys using an aggregate index or score, however, has been limited. Previous metrics for comparing quality across surveys have included *1*) an algorithm-based approach, in which broader dimensions of survey quality, such as selection bias in sampling design and precision of cluster sampling, are rated by individual assessors (9); and *2*) a weighted summative score of 8 different individual indicators of anthropometric data quality based on the Emergency Nutrition Assessment and Standardized Monitoring and Assessment of Relief and Transitions plausibility check tool (8, 22). There are some limitations to both methods for generating a composite measure of quality. An algorithm-based approach, for example, is resource-intensive and subjective because it requires assessment of various dimensions of a survey, beyond those related to anthropometry, by the individual assessor. On the other hand, although an objective measure, a weighted summative score of a large set of individual indicators may include noninformative indicators of anthropometric quality across surveys and may be influenced by indicators with higher variances. The composite index presented in this study has 2 notable improvements over previous efforts. First, we used a data-driven approach to assess item weights based on factor loadings that were derived from the correlation matrix as opposed to assigning weights to individual indicators arbitrarily or letting an individual indicator dominate the score based on its variance (i.e., by using a covariance matrix). Second, we only used individual indicators which were recommended for use in the recent report by the WHO/UNICEF Anthropometry Data Quality Working Group (13). For example, skewness and kurtosis, which have been used previously in other anthropometric quality assessments (6, 8), were not incorporated in the composite index because there is insufficient evidence to suggest that deviation from a Gaussian distribution is due to measurement error alone. The WHO reference population used to derive *z* scores was restricted to a healthy population (23); as such, it is possible that unusual distributions may occur in a population that is more heterogeneous, or in surveys conducted in countries with large population inequities, even in the absence of data quality concerns. The use of HAZ SD and WHZ SD as indicators of quality similarly reflects a combination of measurement error and true population heterogeneity. However, a recent reanalysis of 474 surveys found that the 95^th^ percentiles of HAZ and WHZ SD values were 2.03 and 1.72, respectively, suggesting that some SDs were substantially larger than would be reasonably explained by population heterogeneity alone and therefore more likely reflect issues with data quality (13). While this is consistent with the observation that HAZ SD and WHZ SD had the highest factor loadings in the data quality indices indicating that SD is an important measure of anthropometric data quality, the findings from this study do not provide further insights into the extent to which SD reflects measurement error as opposed to population heterogeneity. In addition, although age and sex ratios have been used to assess data quality by others (24), we opted not to include these in our data quality indices. These ratios can indicate selection bias in either sampling or differences in response rates but in many countries the true relation is not a 1:1 ratio (25). The WHO/UNICEF Anthropometry Working Group recommends comparing survey population age and sex ratios to a reference, such as the UN Population Division World Population Prospects, to distinguish between context-specific natural variation and data quality issues. However, an indicator that needs to be compared against a country's reference population was not viewed as a practical candidate for inclusion in the composite anthropometric data quality index. Furthermore, because The DHS Program has a rigorous sampling process (26), this indicator of quality was less of a concern than anthropometry-specific data quality indicators.

Despite the major strengths of the the composite anthropometry data quality index, we were unable to externally validate the index because gold-standard sources of childhood malnutrition estimates do not exist. However, the face validity of the index is high given that we used a parsimonious and informative set of individual data quality indicators that have been evaluated by a group of experts with international consensus (13), and we found that surveys known to be of high or low quality were ranked accordingly. This is especially apparent in the rare instances in which anthropometry survey results have been suppressed from DHS final reports owing to quality issues (27, 28) or in surveys where data quality concerns were raised, given that different results were reported compared with other surveys conducted at the same time (29). Although not interpretable as an absolute score for external comparisons, the indices effectively rank surveys in relation to anthropometric data quality. We used the first principal component to construct each index, which explains the greatest variance in a single dimension of anthropometric data quality. The total variance explained by each index, however, is relatively low because the individual indicators of anthropometry data quality were not strongly correlated with each other. We favored the easier interpretability of an index that is expressed in a single dimension (akin to the wealth index in the DHS); however, additional principal components could be included to maximize the variability explained by the data quality indices. Furthermore, in instances where distinguishing the different dimensions of anthropometric or broader survey quality is of interest, confirmatory factor analysis may be a more appropriate analytical strategy to reflect the different constructs of survey quality measured by the individual indicators. In addition, in analyses in which the prevalence of stunting or wasting is a covariate of interest, the use of 6Q/4Q data quality indices may complicate interpretation given the correlation of indices with stunting/wasting. In such situations, a restricted anthropometric data quality index, which includes a subset of individual data quality indicators that are unrelated to the location and dispersion of the HAZ/WHZ distribution, may be considered (30). Indicators with relatively low correlation with mean HAZ/WHZ and prevalence of stunting/wasting include the proportion of observations with complete date of birth, proportion of observations with anthropometry measured, digit preference for height, and the absolute difference in HAZ by MOB of reporting.

The composite data quality indices presented in this study are primarily applicable to multisurvey analyses in which measurement of anthropometric data may be assessed across surveys in relative rather than absolute terms. This quality index can be constructed in any set of population-based surveys in which anthropometric data are collected, including the Multiple Cluster Indicator Surveys and national nutrition surveys. Conversely, to assess the quality of anthropometric data in a single survey in isolation, the WHO Anthro Survey Analyser tool provides an automated report on a survey's anthropometry data quality, including indicators which were not included in our derivation of the composite data quality indices (31). Further research on the development of indicators that can better distinguish between data quality problems and sample population heterogeneity is needed to improve the assessment of individual surveys.

Robust anthropometric data quality assessment is vital for monitoring trends in malnutrition among young children and for informing country and global decision-making. The proposed indices of relative anthropometric data quality may be readily derived from the anthropometric data set itself (i.e., external sources of information are not required), and provide a coherent continuous measure for between-survey comparisons that does not rely on the specification of thresholds of high compared with low quality for individual indicators. Although this approach does not evaluate other forms of bias in survey data (e.g., selection bias), the index can be particularly useful in the context of multicountry or time-trend analyses to account for variations in population-level estimates of malnutrition due to heterogeneity in the measurement quality of anthropometric data and to check for robustness of inferences. In addition, it can be used when planning large-scale population-based surveys to highlight key areas for improvement in anthropometry data collection.

## Supplementary Material

nqaa162_Supplementary_Material

## Notes

Supported in part by the United States Agency for International Development (USAID) through The DHS Program, grant #720-OAA-18C-00083 (to SN), the Canadian Institutes of Health Research Sir Frederick Banting and Charles Best Research Award (to NP), and SickKids Child Growth and Development Fellowship (to AA).

Supplemental Figures 1–6 are available from the “Supplementary data” link in the online posting of the article and from the same link in the online table of contents at https://academic.oup.com/ajcn/.

Data described in the article are publicly available upon request from the Demographic and Health Surveys website; the codebook and analytic code will be made available upon request.

Published in a supplement to *The American Journal of Clinical Nutrition*. The Guest Editor for this supplement was Mark Manary, and reports no conflicts of interest. The Supplement Coordinator for the supplement publication was Nadia Akseer, Gates Ventures/Hospital for Sick Children, Toronto, Canada. Supplement Coordinator disclosure: no conflicts of interest. Publication costs for this supplement were defrayed in part by the payment of page charges by Gates Ventures. The opinions expressed in this publication are those of the authors and are not attributable to the sponsors or the publisher, Editor, or Editorial Board of *The American Journal of Clinical Nutrition*.

Abbreviations used: DHS, Demographic and Health Surveys; HAZ, height-for-age *z* score; LMICs, low- and middle-income countries; MOB, month of birth; PCA, principal component factor analysis; WHZ, weight-for-height *z* score; 4Q, 4 data quality indicators; 6Q, 6 data quality indicators.
