# Supplementary material for: Anthropometric data quality assessment in multisurvey studies of child growth
Source: Am J Clin Nutr. 2020 Jul 16;112(Suppl 2):806S–815S. doi: 10.1093/ajcn/nqaa162 (PMC7487428; doi:10.1093/ajcn/nqaa162)
Supplement: nqaa162_Supplementary_Material [file nqaa162_supplementary_material.docx]

**Anthropometric data quality assessment in multi-survey studies of child growth**

Nandita Perumal, Sorrel Namaste, Huma Qamar, Ashley Aimone, Diego G. Bassani, Daniel E. Roth

**Online Supplementary Material**


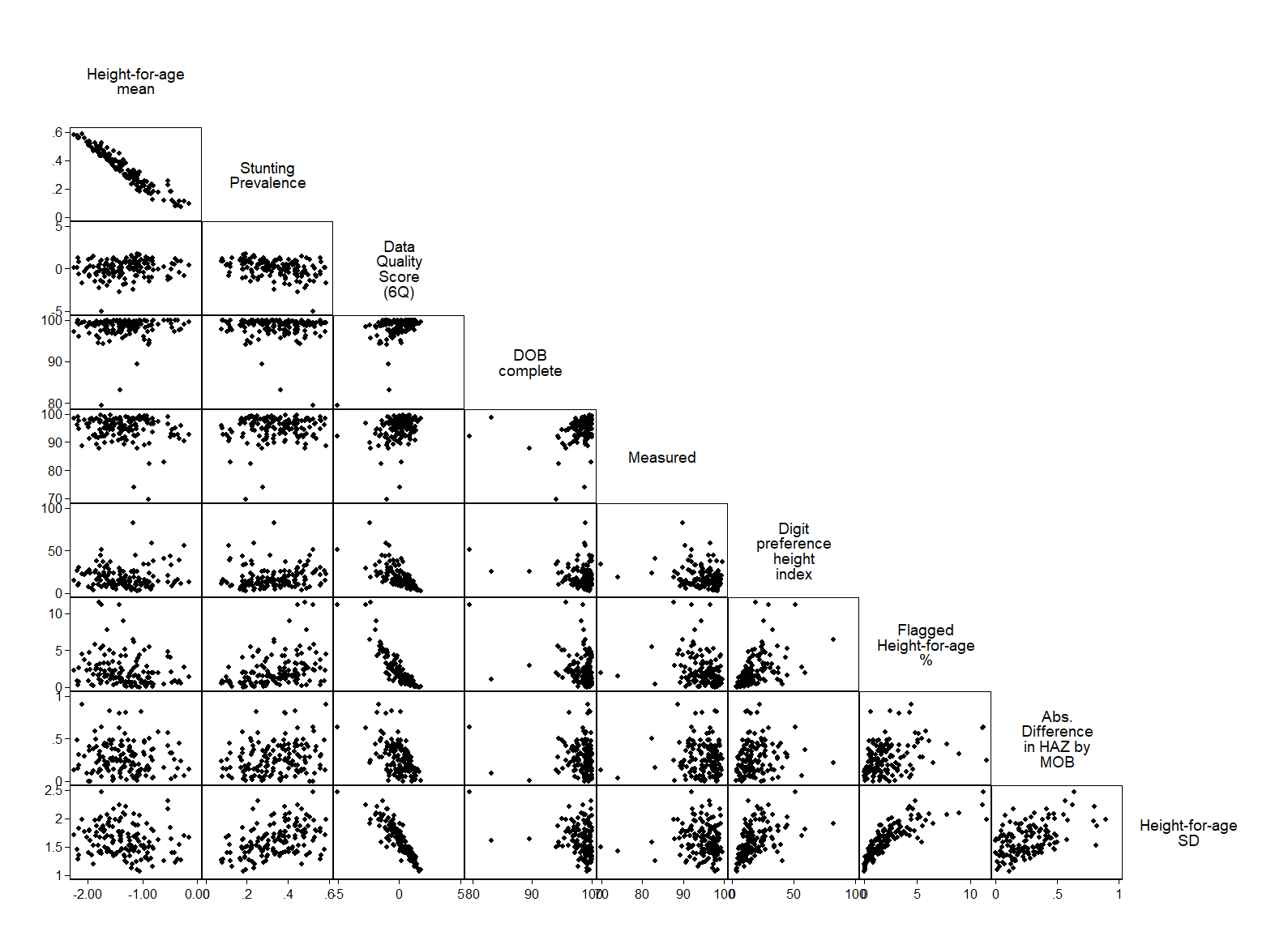


**Supplementary figure 1**. Correlations between mean height-for-age z-score (HAZ), the prevalence of stunting among children <5 years of age, data quality score for HAZ, and the individual indicators of data quality in 145 DHS surveys.


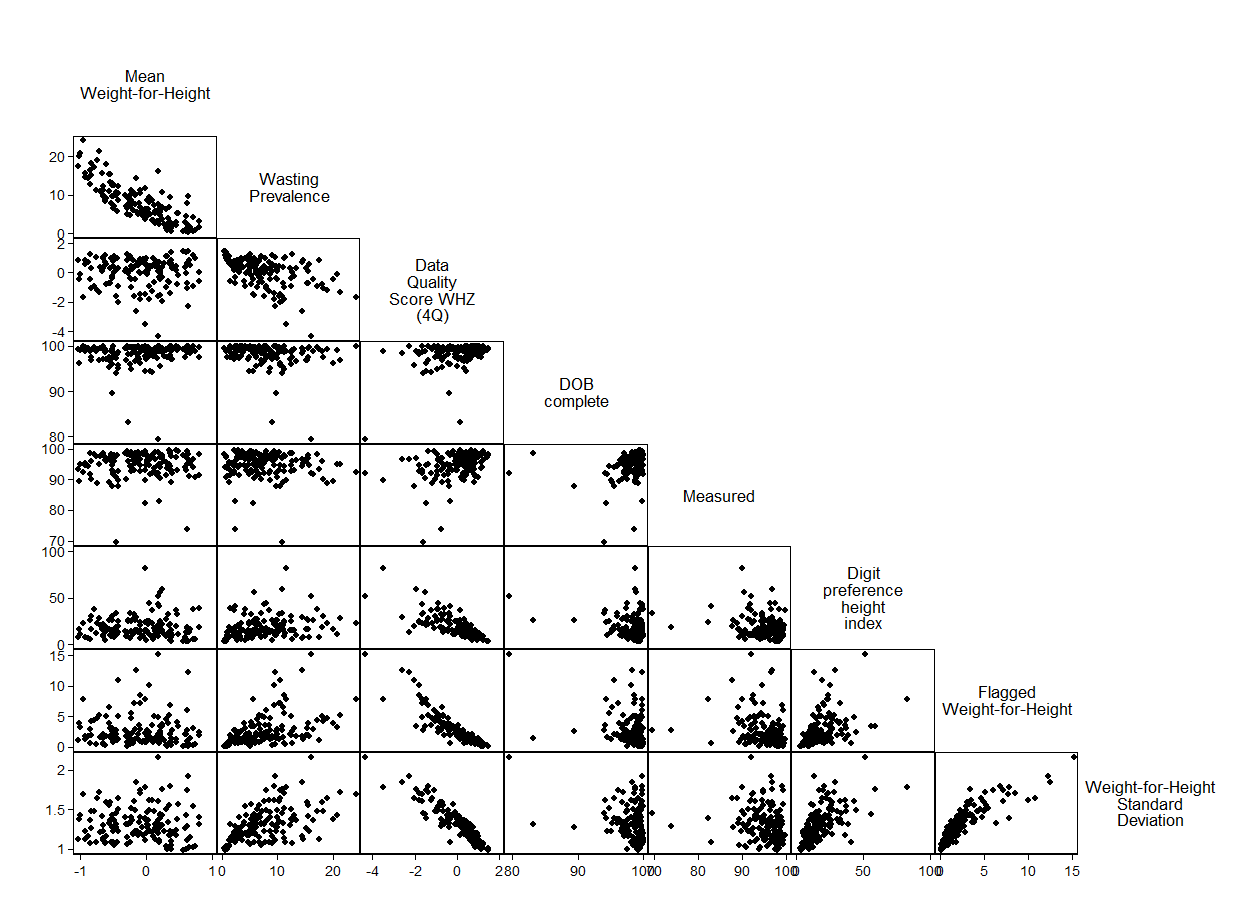


**Supplementary figure 2**. Correlations between mean weight-for-height z-score (WHZ), the prevalence of wasting among children <5 years of age, data quality score for WHZ, and the individual indicators of data quality in 145 DHS surveys.


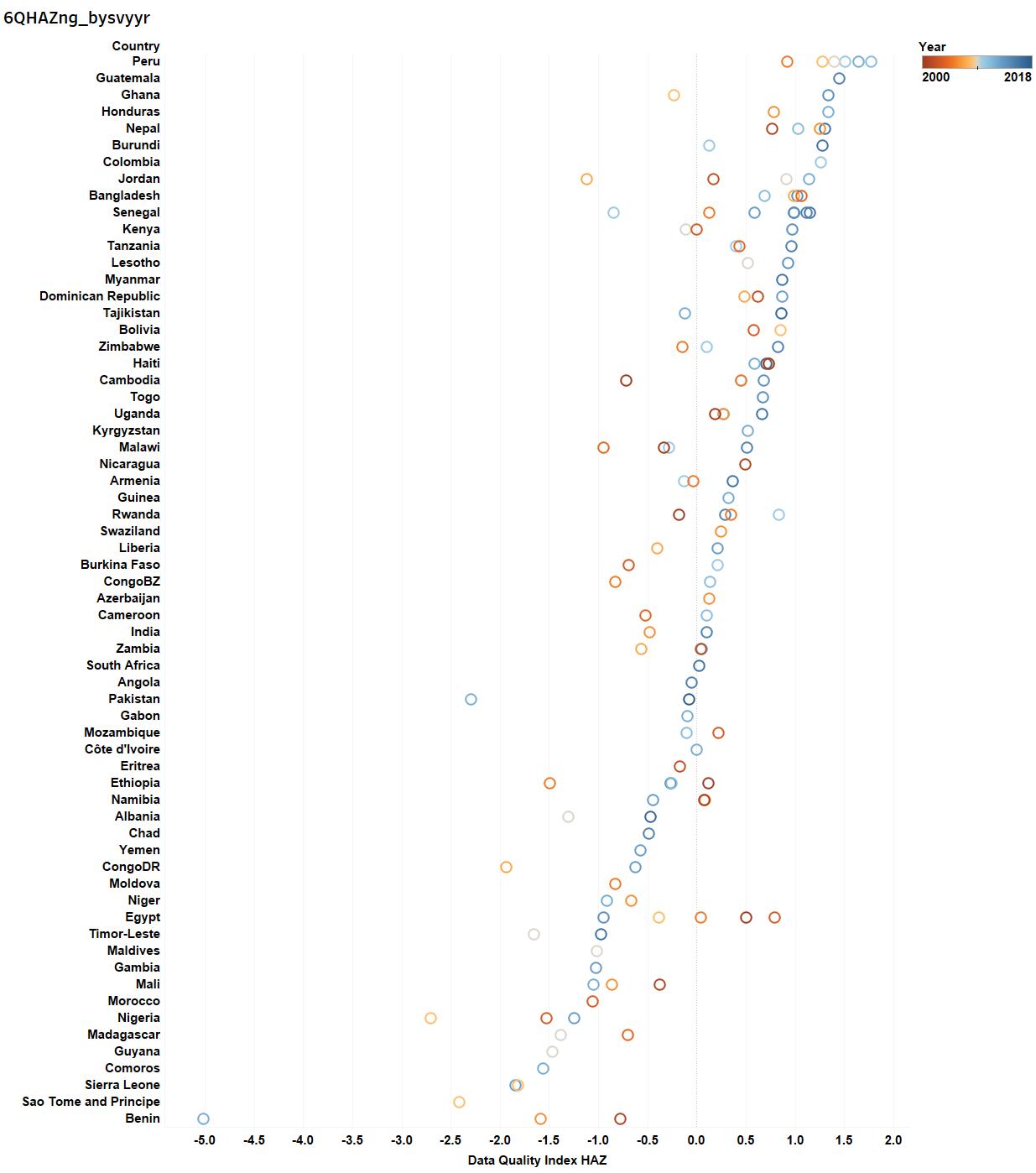


**Supplementary Figure 3**. Country ranking based on anthropometric data quality score for HAZ (6Q) in Demographic Health Surveys by survey year (n=145) over time. Lower score indicates worse anthropometric data quality. Country ranking is based on the data quality score in the most recent survey year.


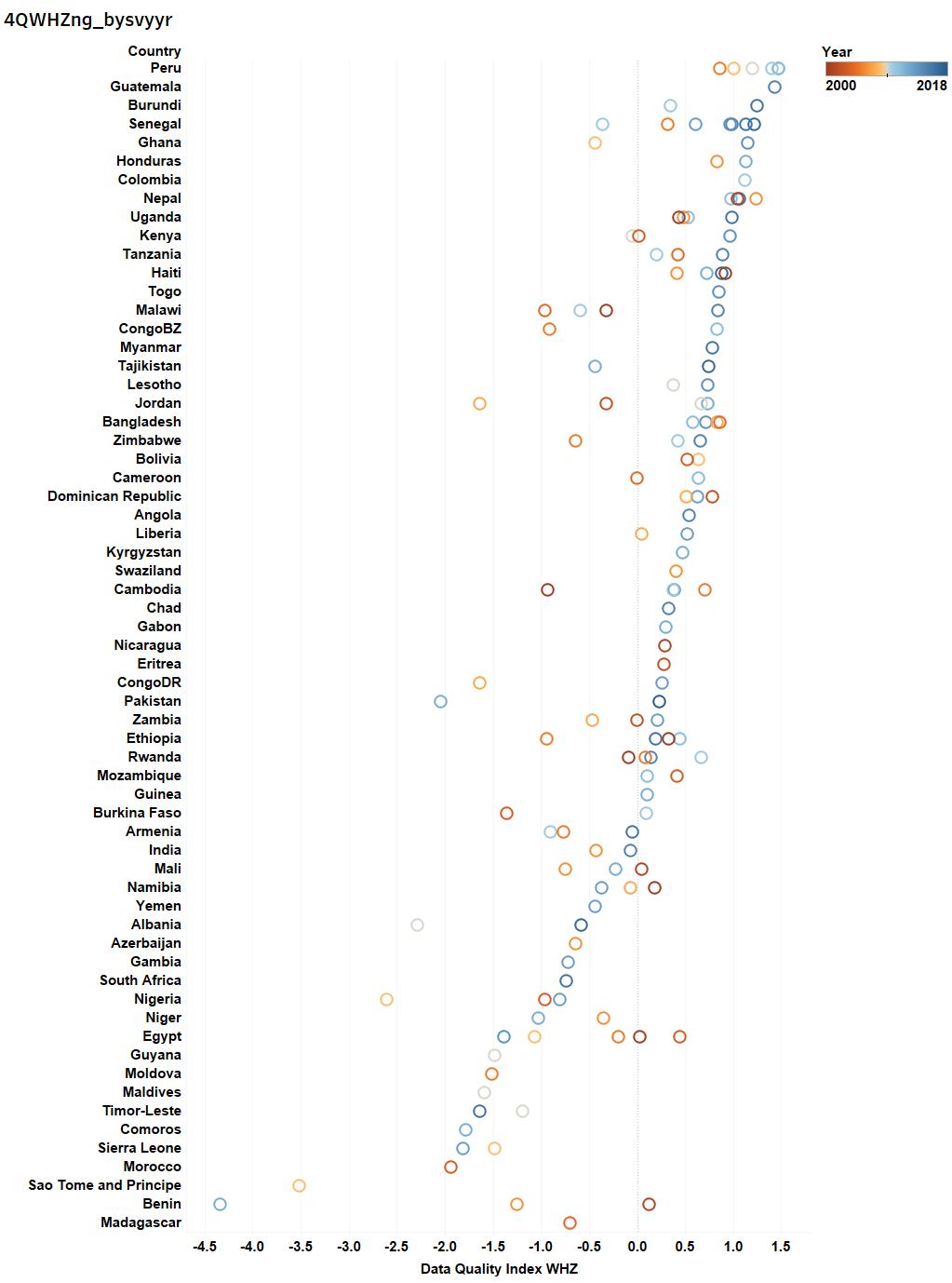


**Supplementary Figure 4**. Country ranking based on anthropometric data quality score for WHZ (4Q) in Demographic Health Surveys by survey year (n=144) over time. Lower score indicates worse anthropometric data quality. Country ranking is based on the data quality score in the most recent survey year.


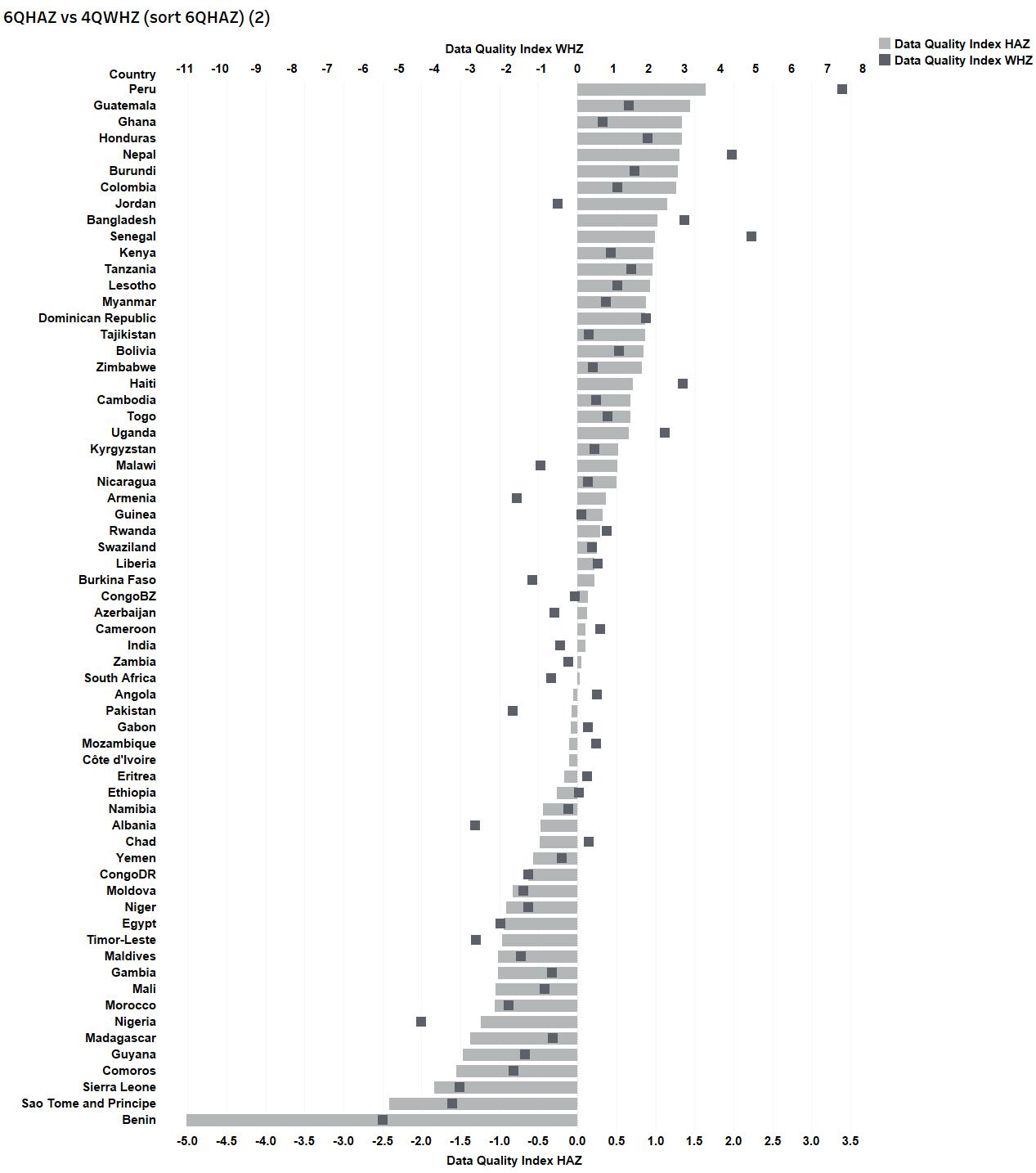


**Supplemental Figure 5**. Country ranking based on anthropometric data quality score for height-for-age z-scores (HAZ) (6Q) for the most recent Demographic Health Survey (n=64) with superimposed data quality scores for weight-for-height (WHZ) (4Q) in dark grey squares. Lower score indicates worse anthropometric data quality.


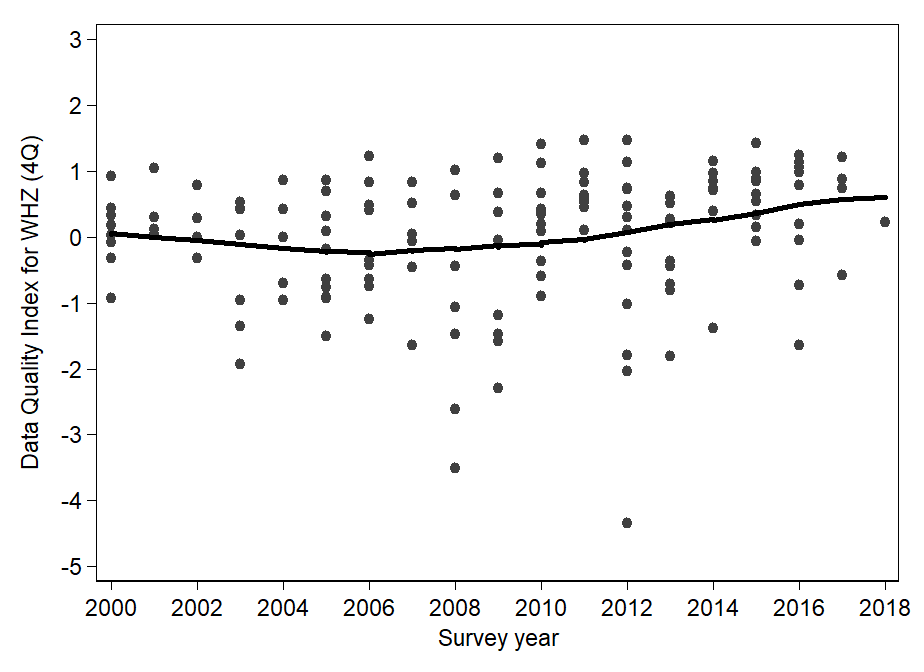


**Supplementary Figure 6**. Scatter plot of anthropometric data quality score for weight-for-height (WHZ) over time in 144 DHS surveys. Change in data quality score over time is shown by the locally weighted smoothing spline.
